# Systematic approach to obtain axillary arterial access for pediatric heart catheterizations

**DOI:** 10.3389/fcvm.2024.1332152

**Published:** 2024-01-31

**Authors:** Raymond N. Haddad, Fatema Karmustaji, Rasha Alloush, Mahmoud Al Soufi, Mohamed Kasem

**Affiliations:** ^1^ M3C-Necker, Necker-Enfants Malades University Hospital, Assistance Publique—Hôpitaux de Paris (AP-HP), Paris, France; ^2^ College of Medicine, Mohammed Bin Rashid University of Medicine and Health Sciences, Dubai, United Arab Emirates; ^3^ Department of Pediatric Cardiology, Heart Centre of Excellence, Al Jalila Children’s Speciality Hospital, Dubai, United Arab Emirates

**Keywords:** axillary access, cardiac catheterization, children, congenital heart disease, transcatheter interventions

## Abstract

**Background:**

Axillary arterial access (AAA) in pediatric heart catheterizations is undervalued.

**Methods:**

We retrospectively reviewed children with congenital heart diseases (CHDs) who received trans-axillary arterial catheterizations between January 2019 and February 2023. We aimed ultrasound-guided punctures in the proximal two-thirds of axillary arteries with diameters ≥2 mm to insert 7 cm/4 Fr short introducers. We administrated intra-arterial verapamil (1.25 mg) and heparin (100 UI/kg). We infiltrated per-operatively 2% lignocaine (10 mg) for arterial spasms, long sheaths use (≥5 Fr), and ≥60 min procedures in <3 kg patients.

**Results:**

We identified 30 patients (66.7% males) with a median age of 1.1 months (IQR, 0.3–5.4), and a median weight of 3.1 kg (IQR, 2.7–3.7). 5/30 patients had six redo interventions after a median of 3.9 months (IQR, 1.7–5.1). Overall, 27/36 procedures were interventional, including 6 aortic valvuloplasties, 6 balloon angioplasties, and 15 stenting procedures. The median arterial axillary angiographic diameter was 2.6 mm (IQR, 2.4–3). Access was right-sided in 23/36 (63.9%) procedures and obtained using 21G/2.5 cm bevel needles in 25/36 (69.4%) procedures. No hemodynamical change occurred after introducing spasmolytic drugs. The median fluoroscopy time was 26.1 min (IQR, 19.2–34.8). There were two self-resolving arterial dissections, one sub-occlusive arterial thrombosis (resolved with 6 weeks of enoxaparin), and one occlusive arterial thrombosis (resolved with alteplase thrombolysis and 6 weeks of enoxaparin). Median follow-up was 11.7 months (IQR, 8–17.5). Four patients with complex univentricular hearts died from non-procedural causes at a median of 40 days (IQR, 31–161) postoperative.

**Conclusion:**

Systematic approach for AAA is the key to success and unlocks the many potentials of trans-axillary pediatric cardiology interventions.

## 1 Introduction

Vascular access is essential for successful diagnostic or interventional cardiac catheterization of newborns and infants with complex or critical congenital heart diseases (CHDs) (1). Axillary arterial access (AAA) is an attractive alternative option for catheter interventions in the pediatric population (2, 3). The AAA could be favorable for procedures like balloon dilation of critical aortic valve stenosis, stenting vertical arterial ducts, opening the struts of previous ductal stents, treating critical aortic coarctation in low birth weight patients, and Blalock-Taussig shunt (BTS) shunt-related interventions (4–13). This is in addition to other cases where femoral arterial access is difficult for many reasons related to low birth weight, vascular abnormalities, or low cardiac output (1–3). However, the AAA is still not routinely used because of the fear of complications as well as the lack of familiarity and experience with this approach. Herein, we present our experience alongside our systematic approach to accessing the axillary arteries in neonates and infants.

## 2 Methods

### 2.1 Study design

We performed a retrospective clinical data review of all children with CHDs who had diagnostic or interventional catheter procedures from the AAA between 01 January 2019 and 27 February 2022. We collected and comprehensively analyzed clinical, procedural, and last follow-up data. We obtained multidisciplinary team approval for each case. We obtained approval from the institutional review board. We obtained signed informed consent from the patient's legal guardians.

### 2.2 Procedure

All procedures were performed by senior operators in a biplane digital catheterization laboratory under general anesthesia and close monitoring. Intravenous antibioprophylaxis was given in stent implantation procedures.

#### 2.2.1 Axillary arterial access technique

We used the right AAA in neonates with critical aortic valve stenosis or critical aortic coarctation and for coronary angiograms as well as interventions on patients with central or right modified Blalock-Taussig shunts (mBTSs). In patients with duct-dependent pulmonary circulation, the site of access depended mainly on the location of the arterial duct and its insertion with the aortic arch. We used the ipsilateral AAA opposite to the duct insertion for stenting an arterial duct originating from the inner curve of the transverse aortic arch. The arch anatomy and the relationship of duct insertion to head-neck arteries were determined by detailed transthoracic ultrasound in most cases. However, we routinely performed CT scans to precisely evaluate the 3-dimensional morphology, tortuosity, length, narrowest as well as widest part, and the pulmonary as well as aortic end of the arterial duct. We also measured the size of the axillary artery on the CT scans.

We placed the child in a normal supine position with the head turned to the contralateral side of the selected AAA side (Supplementary Figure S1). We exposed the axillary area by fixing the arm in a head-up position with a 120–130 degree angle. We avoided additional arm extensions to prevent stretching the vessels and the brachial plexus. We placed the LOGIQ™ *e* ultrasound device (GE HealthCare, USA) on the opposite side of the operator. Under aseptic conditions, we screened the axillary artery with a high-frequency GE L8-18i-D hockey stick probe transducer. We started high in the axilla, with the transducer positioned for a short-axis view, and followed the artery. After identifying the course of the axillary artery, we switched to a long-axis view and obtained a Doppler waveform. We measured the size of the axillary artery and its depth from the skin. We aimed for an artery diameter ≥ 2 mm to accept the 4 Fr/7 cm Prelude® radial sheath introducer—mini access (Merit Medical, USA). We punctured the axillary artery in the proximal two-thirds using a 24G 0.75″ Insyte-W™ yellow intravenous winged catheter (Becton Dickinson, USA) or a 21G × 2.5 cm short green bevel angiographic needle (Merit Medical, USA) without attaching a syringe. We aimed above the branching of the thoracoacromial artery when identified. We performed the puncture in two steps. First, we punctured the skin and aimed to see the needle's bright tip at 12 o'clock on the short-access ultrasound view of the axillary artery. Then, we did a sudden nick of the artery and watched the pulsatile blood back-flow. In case of failure, we pressed on the puncture site to obtain hemostasis. We did not re-use the same winged catheter for more than one puncture. We flushed the needle and used the same one for a second attempt. The puncture was usually done at a 75–85 degree angle with the needle and a 45–55 degree angle with the winged catheter. Both were tilted down almost to skin level after seeing the pulsatile blood back-flow. In both techniques, we inserted and advanced a 0.018″ (0.46 mm) × 40 cm Nitinol wire with a floppy platinum tip under single-plane fluoroscopy. If the wire was in the head or neck vessels, arterial duct, or mBTS, we pulled it back and maneuvered it toward the descending or ascending aorta. We didn't scalp the skin before introducing the sheath. We introduced the short introducer over the wire under fluoroscopy. We kept part of the introducer outside the skin to keep its distal end at the origin of the left or right subclavian artery.

Before proceeding with catheterization, we checked for free blood flow and injected a small amount of contrast solution through the introducer to exclude an intimal lesion or extra-luminal sheath position in the false lumen. We gave an upfront intra-arterial single dose of verapamil chlorhydrate 2.5 mg/ml (1.25 mg) to prevent arterial spasm and heparin (100 UI/kg) to obtain an ACT between 200 and 250 ms. We infiltrated per-operatively 10 mg of subcutaneous 2% (20 mg/ml) lignocaine hydrochloride alongside the verapamil in case of (1) arterial spasm on the opening angiogram, (2) using long guiding sheaths with a size ≥ 5 Fr, and (3) in patients weighing less than 3 kg and undergoing a prolonged procedure (≥60 min). We monitored closely the electrocardiogram rhythm and the blood pressure for any sudden changes. This practice was derived from the adult practice in trans-radial coronary procedures (14, 15). As a part of our nursing protocol, we checked the fingertips every 15 min during the procedure. The introducer tends to get displaced or pushed out during the procedure, especially when inserting catheters, causing access bleeding. Therefore, we manually supported the sheath throughout the catheter procedure. If the introducer is pulled back from the entry point, we insert it back using the dilator.

#### 2.2.2 Ductal stent implantations

During ductal stentings, we routinely used standard or steerable microcatheters to navigate through the ductal curves. We then replaced the floppy 0.018″ wire with a stiffer 0.014″ coronary wire. In the case of an extremely tortuous arterial duct, we used a buddy wire and preferably placed it in a contralateral branch pulmonary artery. The stiff coronary wire was used for stenting. We used drug-eluting coronary stents and aimed to cover the pulmonary ductal end even if the stent migrated a little bit to the proximal section of the branch pulmonary artery. Before stent deployment, we performed angiograms through the introducer sheath. The buddy wire was pulled out before balloon inflation. If it was not done by mistake, we inflated the balloon again to stabilize the stent and pulled out the buddy wire before deflating the balloon. We inflated the coronary stents with saline solution only. The stent is already radiopaque. Adding a contrast medium to the inflation fluid can increase the risk of the balloon under deflation, causing hemodynamic instability and stent migration while pulling the balloon out. We also prevented the tip of the introducer from touching the stent after stent deployment and during the pullback of the balloon inside the sheath. At the end of the ductal stenting procedure, we pulled out the wire after covering it with the microcatheter that we kept in the branch pulmonary artery. This technique prevented the wire from clutching onto the stent struts on the way out. Moreover, the arterial duct is straightened as long as the stiff coronary wire is inside. However, when the wire was retrieved, a sudden southward rotational movement of the ductal aortic end can be encountered, revealing an uncovered ductal tissue towards the aorta. The microcatheter has no straightening power. Therefore, the stent will take its final shape but allow to place a coronary wire through the microcatheter without needing to re-cross the duct in case there is a need for another stent.

### 2.3 Post-operative care

At the end of the procedure, we removed the sheath gently and achieved hemostasis with careful digital compression at the puncture site. We monitored the access area, even when the baby was back in the ward. We looked for changes in skin color, skin warmth, capillary refill, and the quality of the radial pulse as well as changes in the motor activity of the limb. All patients had a routine color Doppler and duplex examination assessment within 24 h post-procedure by senior radiologists, looking for dissection, thrombus, fistula, or aneurysm. We gave continuous intravenous heparin for 24 h overlapped with dual oral antiplatelet therapy (5 mg/kg/day of acetylsalicylic acid and 0.2 mg/kg/day of clopidogrel) only for patients with successful ductal stent placements. Dual antiplatelet therapy was maintained until the second stage surgery. Other patients with uncomplicated AAA did not receive any postoperative anticoagulant or antiplatelet therapy.

### 2.4 Follow-up

Patients' follow-up was performed according to the institutional protocol (clinical examination and transthoracic echocardiography). In all patients, we looked for general and vascular access complications until 3 months of clinical follow-up. Patients with vascular complications had careful surveillance of the vascular access site as well as upper extremity arterial Color Doppler and duplex detailed assessments until vascular patency and normal Doppler flow were confirmed.

### 2.5 Statistical analyses

Statistical analyses were performed using SPSS, Version 22.0 for Macintosh (IBM, Armonk, NY, USA). Categorical variables were reported as frequency and percentage and continuous variables were represented as median with interquartile range. Statistical analysis for continuous variables was conducted using the Mann–Whitney *U*-test.

## 3 Results

### 3.1 Baseline patient characteristics

We identified 30 patients (66.7% males) with a median age of 1.1 months (IQR, 0.3–5.4, range, 0.03–68) and a median weight of 3.1 kg (IQR, 2.7–3.7, range, 2.3–19). Six (20%) patients had an associated genetic syndrome. 5/30 patients had six redo interventional catheterizations from the same AAA after a median of 3.9 months (IQR, 1.7–5.1). The patients' characteristics are outlined in Table 1.

**Table 1:** Patient clinical characteristics.

	*n* = 30 patients
Male gender*, N (%)*	20 (66.7)
Age (months)*, median (IQR)*	1.1 (0.3–5.4)
Weight (kg)*, median (IQR)*	3.1 (2.7–3.7)
Height (cm)*, median (IQR)*	49 (48–55)
Associated genetic syndrome*, N (%)*	6 (20)
Congenital heart disease*, N (%)*	
Univentricular heart program	10 (33.3)
Biventircular heart program	20 (66.7)
Baseline cardiac catheterization procedures*, N (%)*	
Interventional	21 (70)
Balloon aortic valvuloplasty	3 (10)
Balloon angioplasty	3 (10)
Arterial duct stent	1 (3.3)
Aortic coarctation	1 (3.3)
Native right pulmonary artery	1 (3.3)
Stenting procedure	15 (50)
Arterial duct	12 (40)
Aortic coarctation	1 (3.3)
Left pulmonary artery	2 (6.7)
Diagnostic	9 (30)
Aborted attempt to ductal stent (unsuitable anatomy)	1
Coronary angiogram to rule out coronary anomaly	1
Assessment of shunt and pulmonary artery anatomy through central BTS	1
Assessment of pulmonary blood flow source to interrupted right pulmonary artery (likelihood of right arterial duct)	1
Assessment of the repercussion on the ascending aorta during large stenting of a collective chamber in the context of obstructive supra-cardiac TAPVD	1
Assessment of multiple left heart obstructive defects before surgery	2
Pre-Glenn diagnostic catheterization (with aortopulmonary collaterals embolization)	2
Redo-catheter procedures	6 (20)
Balloon aortic valvuloplasty	3 (10)
Balloon angioplasty/ Stented left pulmonary artery	1 (3.3)
Balloon angioplasty/ Right pulmonary artery	1 (3.3)
Balloon angioplasty/ Stented arterial duct	1 (3.3)
Time to redo catheter procedures (months)*, median (IQR)*	3.9 (1.7–5.1)

BTS, Blalock-Taussig shunt; TAPVD, total anomalous pulmonary venous drainage.

### 3.2 Procedure and axillary arterial access

**Figure 1:**
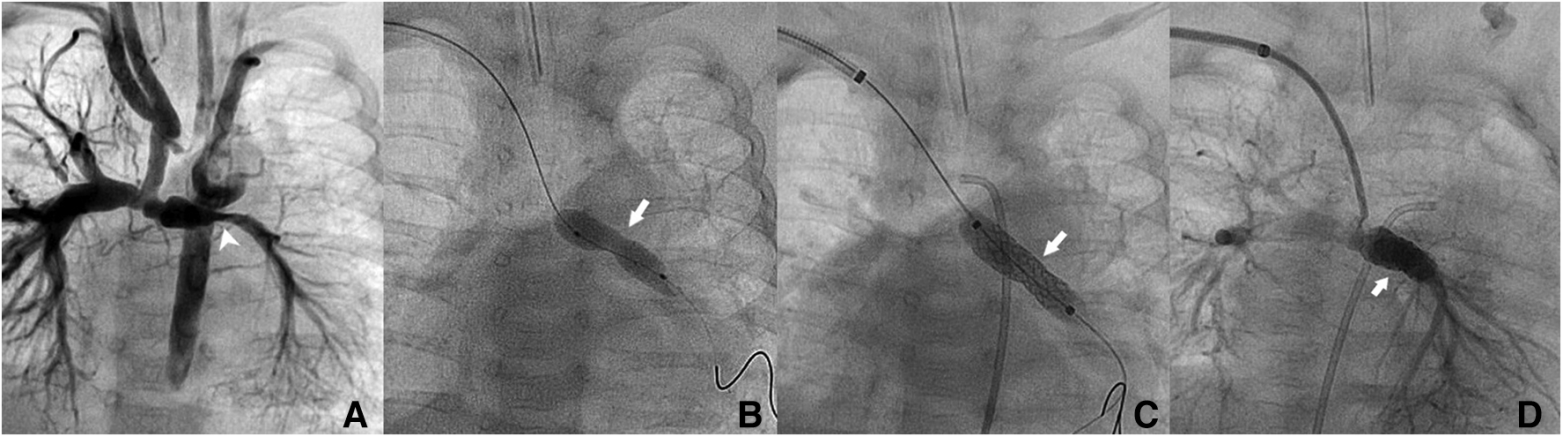
Patient no. 2. Right trans-axillary angiogram showing lumen narrowing of the left pulmonary artery after Norwood procedure in a 2.4-month-old patient (white pointed arrow) (**A**) Dilation with a 5 × 20 mm coronary balloon without waist resolution (white arrow) (**B**) Implantation of a 6 × 18 mm Bentley BeSmooth peripheral stent (**C**) Exit angiogram showing patent left pulmonary artery (**D**).

**Figure 2:**
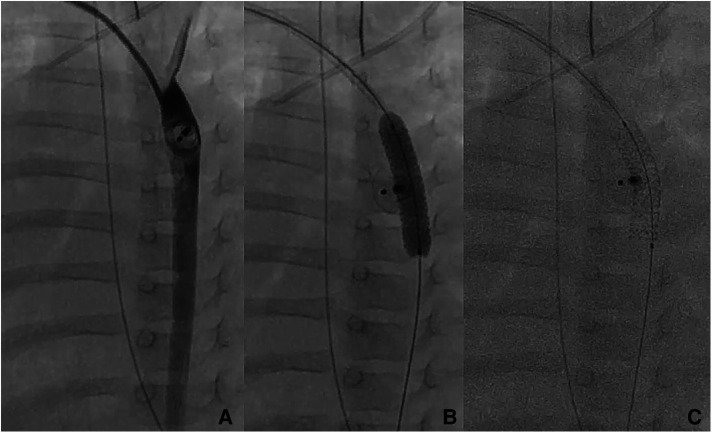
Patient no. 16. Right trans-axillary angiogram showing severe aortic coarctation secondary to ductal device closure in a 6-month-old patient (**A**) Successful implantation on a 5 × 16 mm Boston Scientific Synergy Megatron coronary stent with complete waist resolution (**B,C**).

Overall, 36 catheter procedures were accessed from AAA. 27/36 procedures were interventional, including six aortic valvuloplasties (Supplementary Video S1), six balloon angioplasties, and 15 stenting procedures (Figures 1, 2). Twelve ductal stentings were performed in patients with duct-dependent pulmonary circulation (ten with pulmonary atresia and two with critically obstructed ventricular-pulmonary connection) from the left AAA in eleven and the right AAA in one.

**Figure 3:**
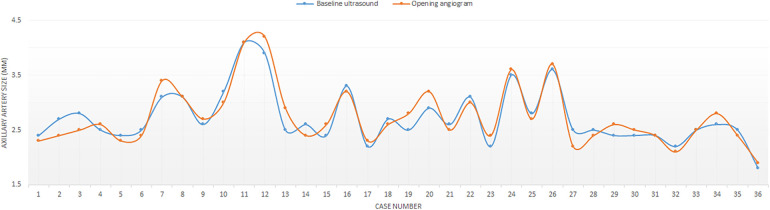
Distribution of arterial axillary size measurements on angiography and ultrasound.

The median angiographic diameter of the arterial axillaries was 2.6 mm (IQR, 2.4–3) and was not significantly different from the ultrasound measurements (*p* = 0.914) (Figure 3). The AAA was right-sided in 23/36 (63.9%) procedures and obtained using short bevel needles in 25/36 (69.4%) procedures. We obtained the AAA from the first attempt in 16 (44.4%) procedures and from the second attempt in 20 (55.6%) procedures. All redo procedures were performed from the same-sided AAA (from the right side in four patients and the left side in one patient). We did not encounter any resistance while inserting the 4 Fr Prelude short introducers. Subcutaneous infiltration of 2% lignocaine hydrochloride (10 mg) was done per-operatively in 8/36 (22.2%) procedures for arterial spasm in one, for the use of a long guiding sheath (≥5 Fr) in two and prolonged procedures in five patients weighing less than 3 kg. We did not observe any hemodynamical change after introducing spasmolytic drugs. We used the 11 cm long 4 Fr Super Sheath™ introducer (Boston Scientific, USA) during four aortic balloon valvuloplasties and one aortic coarctation stenting (Supplementary Video S1). We used one 5 Fr Flexor® sheath (Cook Medical, USA) for a redo aortic valvuloplasty with a 12 × 20 mm Tokai TMP-PED balloon catheter. We used one 6 Fr Flexor® sheath (Cook Medical, USA) to stent the left pulmonary artery using a 6 × 18 mm Bentley BeSmooth peripheral stent (Figure 1). The procedural data is outlined in Table 2.

**Table 2:** Procedure and access related data.

	*n* = 36 procedures
Successful procedures from the AAA*, N (%)*	35 (97.2)
Arterial access switch from axillary to femoral*, N (%)*	1 (2.8)
Pre-procedural CT-scan*, N (%)*	13 (36.1)
Arterial axillary access side*, N (%)*	
Right-side	23 (63.9)
Left-side	13 (36.1)
Arterial axillary puncture method*, N (%)*	
21 G **× **2.5 cm short green bevel angiographic needle (Merit Medical, USA)	25 (69.4)
24 G 0.75″ Insyte-W^TM^ yellow winged catheter (Becton Dickinson, USA)	11 (30.6)
Arterial axillary access introducer size*, N (%)*	
4 Fr (1.3 mm)	34 (94.4)
5 Fr (1.7 mm)	1 (2.8)
6 Fr (2 mm)	1 (2.8)
Arterial axillary access introducer length*, N (%)*	
7 cm (Prelude® radial sheath introducer—mini access, Merit Medical, USA)	31 (86.1)
11 cm (Super Sheath™ introducer, Boston Scientific, USA)	5 (13.9)
Axillary artery echocardiographic size (mm)*, median (IQR)*	2.6 (2.4–3)
Axillary artery angiographic size (mm)*, median (IQR)*	2.6 (2.4–3)
Angulation of branch vessel entry to aortic arch* (degrees)*, median (IQR)*	115 (113–126)
Right axillary artery	117 (114–127)
Left axillary artery	114 (112–118)
Per-operative subcutaneous infiltration of 10 mg of 2% lignocaine hydrochloride	8 (22.2)
Number of puncture attempts*, N (%)*	
First attempt	20 (55.6)
Second attempt	16 (44.4)
Use of long Flexor® introducers (Cook Medical, USA)*, N (%)*	2 (5.6)
5 Fr/70 cm	1 (2.8)
6 Fr/45 cm	1 (2.8)
Microcatheter use*, N (%)*	13 (36.1)
Sheath-in Sheath-out time (min)*, median (IQR)*	90 (78.7–110)
Fluoroscopy time (min)*, median (IQR)*	26.1 (19.2–34.8)
Access-related complications*, N (%)*	7 (19.4)
Medically-treated arterial spasm	1 (2.7)
Self-resolving arterial dissection	2 (5.6)
Sub-occlusive arterial thrombosis	1 (2.7)
Occlusive arterial thrombosis	1 (2.7)
Self-resolving local hematoma	2 (5.6)
Procedural complications*, N (%)*	5 (13.4)
Short run of supraventricular tachycardia	2 (5.6)
Brief low cardiac output state requiring resuscitation	3 (8.3)

We completed successfully 35/36 (97.2%) procedures for the AAA. Patient no.1 (29-day-old, 2.36 kg/48 cm) with pulmonary and tricuspid atresia had a catheter procedure from the left AAA in an attempt to access the arterial duct and stent it. After introducing the 4 Fr short introducer and controlling the access, we observed mild arterial dissection (Supplementary Video S2A) and contrast extravasation (Supplementary Video S2B). The sheath was removed, the access hemostasis was obtained, and the axillary pulse was normal. Subsequent arch angiogram from the femoral artery showed an intact left subclavian artery with no extravasation (Supplementary Video S2C). The arterial duct was extremely tortuous and large, so the stenting procedure was aborted, and the patient was sent for surgical mBTS.

### 3.3 Immediate and short-term outcomes

There were no procedural deaths or permanent procedural complications. There was one arterial spasm that resolved immediately with subcutaneous infiltration of lignocaine and two self-resolving local non-compressive hematomas. There were four more serious access-related complications, including the aforementioned mild arterial dissection. One minor arterial dissection was noticed in a 6-day-old male newborn (2.4 kg/48 cm) on the opening angiogram. The ductal stenting was conducted successfully, and the exit angiogram showed no contrast extravasation. Both patients with arterial dissections did not receive targeted therapy and had normalized vascular ultrasound evaluations on follow-up. One 4.9-month-old female infant (5.7 kg/61 cm) with pulmonary atresia and stenosed ductal stent had successful sequential stent dilation with up-sized balloons from the right AAA. The procedure was challenging and took 2 h. The patient was already taking double antiplatelet therapy. The next day vascular ultrasound showed a sub-occlusive thrombus in the right subclavian artery. The thrombus disappeared within six weeks of enoxaparin therapy. One 3-day-old male newborn (2.3 kg/43 cm) with pulmonary atresia underwent successful ductal stenting from the left AAA. The procedure took 75 min, and the limb pulse was not felt at the end of the intervention. The patient had a pale hand and a discrete change in the limb temperature but without motor deficiency. Urgent vascular ultrasound showed occlusive thrombus. Intravenous thrombolysis with Alteplase was started and overlapped with 24 h of intravenous heparin and then switched to 6 weeks of enoxaparin therapy along with double antiplatelet therapy. The limb pulse was recovered after 8 h. The next day vascular ultrasound showed partial thrombosis, and the vascular patency normalized on day 3 postoperative. A retrospective review of the opening angiogram showed that the axillary artery was borderline and measured 1.9 mm in its mid-proximal section. We did not observe vascular complications during redo procedures.

Overall, there were no permanent vascular complications at 3 months of clinical and ultrasound follow-up. We did not observe any clinical signs or symptoms of nerve injury, motor deficiency, or palmar grasp reflex abnormalities on a median follow-up of 11.7 months (8–17.5). Four patients with univentricular heart physiologies died at a median of 40 days (IQR, 31–161) from the first procedure. All deaths were not procedure-related. One patient with stage-1 palliated hypoplastic left heart syndrome died from severe sepsis. Two patients with pulmonary atresia, intact ventricular septum, and severe coronary artery anomalies had palliative programs and died from coronary occlusions. One patient with pulmonary and tricuspid atresia, large arterial duct, and mBTS died from the complications of severe necrotizing enterocolitis. The patient outcomes are outlined in Table 3.

**Table 3:** Immediate and follow-up patient outcomes.

	*n* = 30 patients
Antiplatelet therapy regimen*, N (%)*	
None	9 (30)
Acetylsalicylic acid	5 (16.7)
Clopidogrel	–
Acetylsalicylic acid + Clopidogrel	16 (53.3)
Intensive care unit stay duration (days)*, median (IQR)*	8 (2–24)
Hospital stay duration (days)*, median (IQR)*	14 (8–43)
Follow-up duration (months)*, median (IQR)*	11.7 (8–17.5)
Late non-procedural-related death*, N (%)*	4 (13.3)

## 4 Discussion

We present our successful experience alongside our systematic approach to accessing the axillary arteries in 30 small children. We demonstrate that the AAA is a safe and attractive approach for pediatric cardiology interventions with several clinical and procedural advantages.

### 4.1 Clinical and procedural benefits

The AAA for catheter procedures in neonates and infants with CHDs is a real alternative approach to the more commonly applied femoral or even carotid artery access. The most interesting clinical advantage of this AAA is that the axillary artery is not an end-artery (2, 4, 16). The arm continues to be perfused by the second intercostal and acromial arteries while the axillary artery is cannulated. Two categories of patients with CHDs that are better treated from the AAA are neonates with critical aortic valve stenosis with or without aortic coarctation as well as those with duct-dependent pulmonary circulation and a tortuous arterial duct originating from the inner curve of the aortic arch opposite to the origin of the left or the right subclavian artery (2–13). The axillary artery is felt better than the femoral artery in low birth weight newborns, particularly in those suffering from critical aortic coarctation with non-palpable femoral pulses. In these patients, the use of femoral arterial access has a high risk of significant vessel damage due to low cardiac output and impaired flow to the lower part of the body (4–10). The carotid artery approach, whether surgical or percutaneous, is another alternative with many reported technical benefits and promising outcomes (17–19). However, in the case of thrombus formation, the AAA is potentially superior to carotid access which exposes the patient to a higher risk of thromboembolic stroke.

Ductal stenting as a minimally invasive alternative to mBTS is being increasingly performed in neonates with duct-dependent pulmonary circulations (3, 12, 13). However, in neonates with severe right heart obstructive lesions early in fetal life, the arterial duct is long, tortuous in different planes, originates frequently from the inner curve of the transverse aortic arch, and presents with an overall vertical course (2, 3). In those ductal anatomies, the retrograde femoral artery route is challenging but is feasible using a 4 Fr Judkins left coronary catheter or a 4 Fr pigtail catheter with its loop cut to give an “inverted-J shape”. Both catheters can be used to engage the aortic ductal ampulla and catheterize the duct. However, it is more difficult to secure a stable wire position for tracking the balloon-stent unit. It is also possible to access the aorta anterogradely from the femoral vein through the heart utilizing a Judkins right catheter, but this route makes the control of the catheter more difficult and may cause hemodynamic instability in small babies by keeping the atrioventricular and semilunar valves open. Polat TB showed that fluoroscopy time and procedural time were significantly shorter in patients in whom the ductal stenting was done from the AAA in comparison to those who had anterograde procedures from the femoral vein (20).

### 4.2 Technical aspects and considerations

Surgical cut-down of the AAA has been reported in infants and children (21). However, this approach is challenging for the surgeons because the axillary artery is surrounded by little soft tissue, and the nerves around the vessel could get damaged during the cut-down. Viswanathan S et al. examined the long-term vascular sequelae of AAA and demonstrated flow abnormalities on vascular ultrasound which were of uncertain clinical significance (21). We, however, with our approach did not encounter any sign or symptom of nerve injury or vascular flow abnormalities on 3 months of follow-up. Using a needle or a winged catheter to obtain the AAA was based purely on the operator's preference and usual practice. We did not observe any advantages or limitations using one material or another. We also did not find that complications were more frequently associated with one puncture hardware.

The 4 Fr short introducer is sufficient for most interventions in babies as it can accommodate most coronary stents and balloons. Although they recommend a 5 Fr size on the stent cover, this is meant for the guide catheter instead of the short sheath introducer. During balloon dilation of critical aortic valve stenosis, we discovered the utility of the 11 cm long 4 Fr Boston Scientific adult radial introducer. The tip of the sheath can reach the aortic root and support the back of the balloon during inflation. This will minimize the balloon pushback from the aortic valve level during inflation and eliminate the need for multiple inflation attempts. This practice also eliminates the need for rapid pacing or intravenous adenosine to stabilize the balloon during inflation. The drawback of this approach is the sheath exchange. Inserting a 4 Fr adult radial sheath introducer requires a 0.035″ wire. We still prefer to insert the Merit Medical Prelude pediatric introducer first and then exchange it with the adult sheath over a 0.035″ wire. The alternative option is to insert the 4 Fr adult introducer directly on the 0.018″ wire of the pediatric introducer. However, the dead space between the introducer and the wire can damage the arterial wall. Consequently, we abstain from doing this.

Pediatric axillary artery cannulation can be challenging because of the small vessel size. Most experts in percutaneous coronary procedures agree on the need for prophylactic spasmolytic medications to reduce radial artery spasms and the potential risk for procedural failure. The current evidence demonstrates the benefit of using vasodilators to reduce radial vasospasm and patient discomfort (14, 15). However, there is still an inconsistency in the literature regarding the preferred medication and the most effective spasmolytic regimen (22–24). We adopted this adult practice and customized it for our pediatric population. Various vasodilatory medications and combinations have been tested recently in pediatric patients and have shown an improved first-attempt success rate and reduced the complication of radial artery catheterization under ultrasound guidance (25, 26). We hypothesized that our spasmolytic regimen would improve the success rate of pediatric axillary artery cannulation. We felt safer increasing the internal diameter of the axillary artery and limiting its susceptibility to spasm after sheath insertion. This approach appeared clinically effective as we observed one arterial spasm on the opening angiogram and did not encounter any resistance while inserting the short introducers. The safety of our spasmolytic regimen was also demonstrated in this series because we did not observe any clinical adverse events.

Manual access hemostasis is one of the most important tasks and should be performed gently and patiently by senior operators for at least 30 min. Generous compression may be complicated with vascular thrombosis, especially in small newborns. Puncturing the axillary artery in the first or second proximal thirds allows a more effective vascular compression against the second rib and avoids many of the branch vessels. Injury to the brachial plexus is also more easily avoided in this region as the nerve bundle travels cephalad to the axillary artery (27).

### 4.3 Drawbacks and limitations

Obtaining the AAA can be challenging for some operators not familiar with the upper arm vasculature. We believe that successful AAA is conditioned by careful preparation of the axillary area and the proper use of Doppler-guided puncture. First, advancing the short needle or the winged catheter with infirm and short jabs, rather than with a continuous and slow motion avoids dislocation of the axillary artery, as it is surrounded only by soft tissues. Second, real-time imaging of the needle entering the vessel requires hand-eye coordination and proper training (28). We have developed that skill through practice and systematic use of ultrasound guidance for all vascular access. Third, the procedure must be performed on a well-sedated or intubated patient in whom the upper limb is placed in a head-up position. We did not attempt any blind puncture, even though it has been reported as feasible by others (2). There are no other anatomical landmarks available for a blind puncture if the axillary pulse is not felt well. Generous infiltration of local anesthetics may increase the depth of the axillary artery and alter the anatomical landmarks (2).

The left AAA is more difficult than the right because right-handed operators are unfamiliar with standing on the left side and the equipment setting can be a little cumbersome. Therefore, it is advisable to have four rather than two experienced hands. In this setting, the “flip technique” with the patient reoriented on the catheterization table can enhance procedural simplicity as reported by Bauser-Heaton H and his colleagues (3). In elective procedures, we think that if the AAA is not obtained from the second attempt, it is advisable to stop the intervention and try a couple of days later, reducing the odds of vascular injuries. The axillary approach is not risk-free and potential complications may occur as in any other arterial access (29–31). We observed that serious vascular complications occurred mainly in very low birth weight neonates with borderline-sized axillary arteries. In these small babies, prolonged interventions increase the burden and manual hemostasis is extremely challenging as the flow can be easily disrupted even during gentle compression. However, all complications were resolved with proper management and no patient experienced permanent sequelae. Therefore, we are convinced that with increasing experience and a systematic approach to obtaining the AAA, complications can be minimized and even avoided.

## 5 Conclusions

We present our successful experience alongside our systematic approach to cannulate axillary arteries in neonates and infants. We introduce a new spasmolytic regimen, which in our series showed to be safe and effective, as a safe option to prevent axillary artery spasms and potential complications. We conclude that AAA is a safe and attractive approach for pediatric cardiology interventions with several clinical and procedural advantages.

## Data Availability

The data analyzed in this study is subject to the following licenses/restrictions: The raw data supporting the conclusions of this article will be made available by the authors, upon reasonable request, to any qualified researcher. Requests to access these datasets should be directed to raymondhaddad@live.com.

## Supplementary material

Supplementary Figure S1 Patient positioning on the catheterization table for left AAA.

Supplementary Video S1 Patient no. 4. Right trans-axillary angiogram through an 11cm long 4 Fr Super Sheath™ introducer (Boston Scientific, USA) shows aortic valve stenosis in one-month-old patient (A). Balloon valvuloplasty (B). Exit angiogram showing no aortic valve insufficiency (C).

Supplementary Video S2 Patient no. 1. Mild arterial dissection and contrast extravasation on opening angiogram from the left AAA (A, B). Subsequent transfemoral arch angiogram showed an intact left subclavian artery (C).
