# Supplementary figures and images for: Systematic approach to obtain axillary arterial access for pediatric heart catheterizations
Source: Front Cardiovasc Med. 2024 Jan 31;11:1332152. doi: 10.3389/fcvm.2024.1332152 (PMC10864575; doi:10.3389/fcvm.2024.1332152)

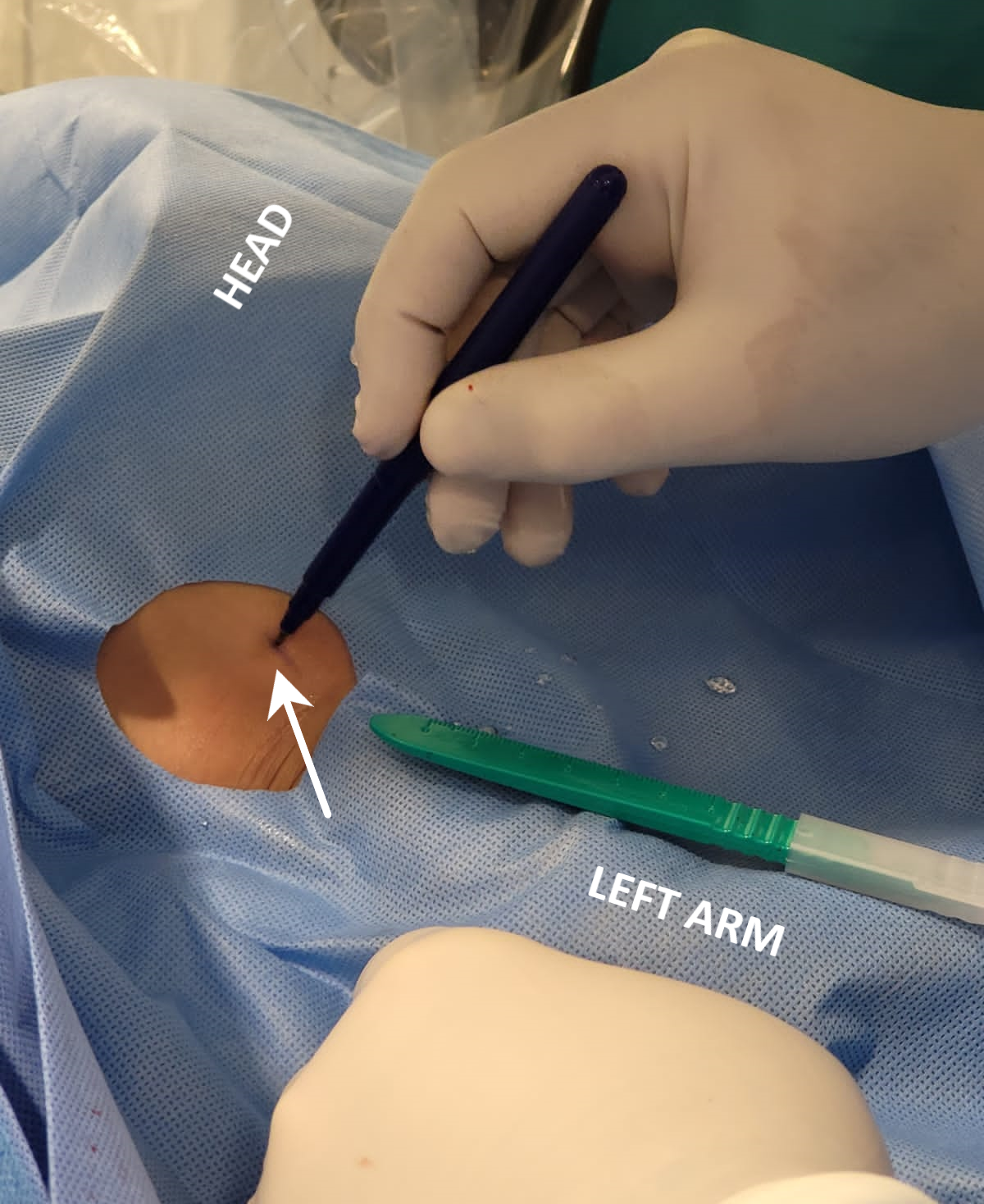

Supplement: Supplementary Figure S1 [file Image1.tif]
